# Tumor PD-L1 Induction by Resveratrol/Piceatannol May Function as a Search, Enhance, and Engage (“SEE”) Signal to Facilitate the Elimination of “Cold, Non-Responsive” Low PD-L1-Expressing Tumors by PD-L1 Blockade

**DOI:** 10.3390/ijms20235969

**Published:** 2019-11-27

**Authors:** Tze-Chen Hsieh, Joseph M. Wu

**Affiliations:** Department of Biochemistry and Molecular Biology, New York Medical College, Room 147, Valhalla, NY 10595, USA; tze-chen_hsieh@nymc.edu

**Keywords:** dietary polyphenols, resveratrol, piceatannol, PD-L1

## Abstract

Programmed cell death ligand 1 (PD-L1) is an immune regulatory protein that facilitates tumor escape from host immune surveillance. In the clinic, tumors with high level of PD-L1 have been used to identify patients who might respond favorably to treatment by anti-PD-L1 antibodies (PD-L1 blockade, PLB). Typically, a progression-free response of 9–20% to PLB has been observed, the basis for the low success rate is largely unknown. Recently, we show upregulation of PD-L1 in cancer cells by ≥IC_50_ supra-pharmacological dose of grape polyphenol resveratrol and piceatannol, alone and combined. Herein, we summarize recent published studies on the regulation of tumor PD-L1 by flavonoids and grape polyphenols. We hypothesize that the induced tumor PD-L1 by resveratrol and/or piceatannol may serve as a Search, Enhance, and Engage (“SEE”) signal to sensitize and augment the recognition and detection of low PD-L1-expressing “cold, non-responsive” tumors. The “SEE” strategy enhances the “visibility” of previously unidentified tumor cells for targeting and eventual eradication by the host antitumor activity. This strategy expands the selection criteria for patients with improved sensitivity and potential responsiveness when used in combination with PLB. The modulation of tumor PD-L1 by flavonoids or polyphenols is proposed to improve the response to PLB in low PD-L1 tumors.

## 1. Introduction

The PD-L1 is an immunoregulatory protein that has varying level of expression in immune and tumor cells [1,2]. PD-L1 plays a functional role in maintaining immune homeostasis and facilitating immune escape. Physiologically, binding of PD-L1 to its receptor program death 1 (PD-1) on T cells constitutes a “safety brake” to prevent over-reactive host immunity, whereas, pathologically, in cancer cells, aberrant expression of PD-L1 and its binding to cytotoxic T cells act as a “stop sign” to counter the proliferation and function of T cells, thus augmenting the tumor evasion of host anti-tumor immunity. 

In recent years, remarkable success has been observed in numerous human immune-oncology trials using an approach widely referred to as PD-1/PD-L1 immune checkpoint blockade (PLB). PLB engenders specific monoclonal antibodies to disrupt the interaction between PD-L1-expressing tumor cells and PD-1-expressing cytotoxic T cells to block the defensive shield tumor cells utilize to incapacitate T cells in the tumor microenvironment. This approach effectively counteracts the inactivation of host T cells by tumor cells and facilitates tumor cell elimination by host immune cells. As such, PLB differs from traditional chemotherapies: its anti-tumorigenic prowess lies in forestalling the host tumor immune surveillance by modulating and igniting the expression and activity of the repertoire of cytotoxic T-cell and T-regulatory cells. Clinical efficacy of this approach has been shown in multiple cancer types including melanoma, lung, renal and breast carcinomas [3,4,5,6].

Although the advent of anti-PD-1/PD-L1 therapy has made it possible to achieve tumor eradication, disease remission and cure; clinically, however, the life-prolonging effectiveness of this approach is limited to a relatively small percentage (<20–30%) of patients, typically in individuals expressing high PD-L1 tumors. Several factors appear to be crucial for the efficacy of PLB. An increase in cancer cell PD-L1 expression is correlated with the decrease in proliferation and increase in apoptosis of T cells, and evasion of the host immune surveillance [2,7]. Moreover, successful clinical outcome to PLB may be linked to the specificity and potency of the PD-1 and/or PD-L1 blockade monoclonal antibodies administered to patients [8,9,10,11,12,13]. From the mechanistic and therapeutic perspective, both factors are relevant to PLB since they are integral to the formation of the molecular bridge that connects receptor-ligand interaction existing between the immune and cancer cells for driving T cell proliferation and subsuming tumor cell escape from host immune surveillance. Therefore, gaining an understanding of the control of PD-L1 in cancer is imperative, to improve efficacy of existing PLB, and for the development of future generation PLB. Knowledge of how PD-L1 is regulated in tumor cells is also essential for tackling open questions in PLB, such as why progression-free response to PLB is largely limited to patients expressing high PD-L1, and whether tumor PD-L1 is merely a biomarker for differential response to PLB. Other unresolved issues of clinical interest and relevance include: What governs the expression of the hyper-elevated PD-L1 in tumorigenesis? Can PLB-responders be identified with certainty using biochemical profiles/markers, distinct of PD-L1? What genetic, molecular and immunogenic profile changes characterize the PLB-non-responders? Do such profile changes have clinical and therapeutic significance and implication, and can they be studied using tissue cultured cell models?

Resveratrol is a grape polyphenol first reported to exhibit chemoprevention activity in 1997 [14]; in the ensuing years, voluminous scientific publications appeared showing its health benefits in a wide range of biological systems [15,16]. Previously resveratrol was shown to induce IFN-γ expression in CD8+ T cells both ex vivo and in vivo [17], and also to inhibit CD4+ T cells [17,18]. These results suggest that resveratrol modulates host immunity; however, whether it regulates the expression of immune checkpoint regulatory molecule, PD-L1 in cancer patients has not been investigated. This unanswered question is of clinical significance since, as discussed above, high tumor PD-L1 expression is correlated with an efficacious, durable, clinical outcome in patients treated with anti-PD-L1-based PLB. Conceivably, it may be proposed that an alteration of tumor PD-L1 expression by diet-derived polyphenols could impact the patient response to PLB. Of interest, whereas we showed upregulation of PD-L1 in several types of cancer cells by ≥IC50 supra-pharmacological dose of grape polyphenol resveratrol and piceatannol, alone and combined [19], a contra-indicated result was obtained in PubMed search demonstrating that in in vitro studies, expression of tumor PD-L1 is suppressed by bio-achievable, pharmacologic dose of flavonoid polyphenols. These studies on flavonoids and on resveratrol and piceatannol in control of tumor PD-L1 are hereby summarized. We propose that upregulation of tumor PD-L1 induced by resveratrol/piceatannol may serve to flag or sensitize the tumor cells for targeting by PLB through an increase in tumor cell “visibility”. By contrast, suppression of tumor PD-L1 by flavonoid polyphenols may decrease tumor cell “visibility”, thus possibly reducing the required therapeutic dose and, in parallel, the side effects of PLB. Our hypothesis implies that the beneficial response rate to PLB alone or in combination with other therapeutic regimens may be enhanced or attenuated using dietary polyphenols. 

## 2. Suppression of PD-L1 Expression by Bio-Achievable Pharmacologic Concentration of Flavonoid Polyphenols

The seminal report of Doll and Peto in 1981 documenting the summative evidence that an association exists between nutritional factors with various common human cancer types laid the foundation for the voluminous in vitro, animal and epidemiological studies performed in diverse geographic locations in the ensuing decades [20]. That diet and nutrition are causally related to the incidence and mortality of cancers in humans also spearheaded the shift in research, starting in 1990s, for the identification and characterization of specific, purified dietary ingredients with a contributory or participatory role in conferring protection against human malignancies. Among agents identified are flavonoids and polyphenols, which have been extensively investigated for cancer prevention. These structurally diverse, polyphenolic compounds are present in abundance in fruits, vegetables, and plant-derived beverages such as red wine, tea, and in food components frequently consumed by the public. The daily intake of flavonoids in an average U.S. diet has been estimated to be in the neighborhood of 0.2–1.0 g while the maximum plasma concentration of a specific flavonoid or group of flavonoids rarely exceeds 10 μM post ingestion [21,22,23,24]. However, in vegetarians and dieters who may consume more fresh fruits, vegetables and soy products and in individuals taking flavonoid-rich nutritional supplements, a significantly higher endogenous level of flavonoids may be achieved [25,26,27]. Both in vitro cell culture studies and animal experiments show that flavonoids can inhibit cancer initiation and progression, and that the effects are compound-specific [28,29,30,31,32]. Cancer prevention activities have been demonstrated for several flavonoids, predominantly genistein and quercetin, considered the major flavonoids consumed in an typical American diet [25]. Notably, at doses tested in cultured cell studies, most flavonoids show selective killing of cancer cells over healthy, normal cells [33].

To investigate the health-promoting immunotherapeutic role of diet-derived flavonoids and polyphenols, we performed a PubMed search. A query of “immunomodulation and flavonoids” yielded 375 output while combining “flavonoids and immunotherapy” resulted in 192 hits. For comparison, a search using “polyphenols and immunomodulation” or “polyphenols and immunotherapy” retrieved 88 and 48 scientific articles of interest. A targeted search using “resveratrol and PD-L1” or “flavonoids and PD-L1” provided 13 hits. The studies demonstrating the most definitive results and conclusions with respect to the regulation of PD-L1 are presented in Table 1. It shows that in in vitro experiments using cultured cancer cells, various flavonoids effectively suppress IFN-γ or EGF mediated induction of PD-L1 [34,35,36]. As illustration, IFN-γ-induced PD-L1 at the protein level was significantly and variably upregulated in human melanoma A375 (2-fold), A2058 (3-fold) and RPMI-7951 (4-fold) cells, respectively. The increase in PD-L1 in A375 and A2058 tumor cells was attenuated by the addition of 25 µM curcumin and almost completely suppressed by 30 µM apigenin. The observed inhibition of PD-L1 by curcumin or apigenin was coordinated with the inhibition of signal transducer and activator of transcription 1 (STAT1)—a transcription mediator of PD-L1. These results suggest that in these two melanoma cell lines attenuation of IFN-γ-mediated induction of PD-L1 by both flavonoids occurs by affecting PD-L1 transcription. However, a different mechanism most likely operates in RPMI-7981 tumor cells in which inhibition of PD-L1 protein expression by curcumin or apigenin was substantially less pronounced compared to the corresponding change in expression of STAT-1, implying that a complex mechanism of regulation of PD-L1 is involved. Although the doses of curcumin or apigenin used in these experiments exceed 10 μM flavonoid dose typically found in humans post ingestion [21,22,23,24], they may be achieved in individuals after intake of flavonoid-containing supplements and in vegetarians who may consume large quantities of fresh fruits, vegetables and soy products [25,26,27]. It should also be noted that, at doses studied, both apigenin and curcumin cause G2/M cell cycle blockade as well as induction of apoptosis in melanoma cells, but show little anti-cellular effects in normal melanocytes [34]. In a separate study, it was observed that induction of PD-L1, both at protein and mRNA levels, by IFN-γ or EGF in human non-small cell lung carcinoma cells were correspondingly attenuated by exposure to green tea catechin EGCG and several other tea catechins [35]. Similarly, Ke et al. [36] showed that baicalein, a flavonoid found in *Scutellaria baicalensis* Georgi, dose-dependently (2.5, 5, or 10 µM) inhibited by ~60–70% IFN-γ (10 ng/mL) mediated induction of PD-L1 in hepatocellular carcinoma SMMC-7721 and HepG2 cells [36]. Ke et al. [36] also reported that the therapeutic effectiveness of baicalein persisted in T-cell-deficient mice, counteracting the significance of the down regulation of PD-L1 by flavonoids isolated from *Scutellaria baicalensis Georgi*. However, other plausible explanations relevant to the observed results also should be considered. A close examination of the studies of Ke et al. [36] suggests that the effects of baicalein was more pronounced on membrane surface expression of PD-L1 assayed by flow cytometry, than determination of total cellular expression of PD-L1 by western blotting, suggesting that baicalein might affect distinct pools of PD-L1 targeted for differential control of immune escape versus regulation of anti-tumor immunity in the tumor microenvironment. A different consideration stems from recent report by Poggio et al. [37] showing that removal of exosomal PD-L1 inhibits tumor growth and that exposure to exosomal PD-L1-deficient tumor cells suppresses growth of wild-type tumor cells injected at a distant site, simultaneously or months later. Also noteworthy in regard to Ke et al. [36] results is the anti-cancer immunological role of B cells. Thus, experiments have shown that B-cell subpopulations are first recruited to the tumor site followed by acquisition of immunosuppressive activity, concomitant with the attenuation of their anti-tumor immune response within the tumor bed [38,39]. Accordingly, whether B cell anti-tumor immunologic activities can be modulated by baicalein and its derivatives are open, unresolved questions that must be further investigated in future studies. We additionally proffer that flavonoid polyphenols might control the proliferation and function of the myeloid-derived suppressor cells (MDSCs) which contribute to tumor-mediated immune escape and negatively correlate with overall survival of cancer patients. Exploratory studies are underway to test the effectiveness of flavonoids to suppress the expansion and function of MDSCs as a combat strategy to increase immunotherapy efficacy [40,41,42].

Clearly, numerous other unanswered questions remain. For example, can co-treatment with combinations of flavonoids lead to more effective, synergistic suppression of PD-L1 expression, by down regulation of STAT-1 as well as by inhibition of the activation of NF-κB—both required for transcription control of PD-L1—and accordingly diminish the propensity of cancer cells to exhibit escape from immune surveillance, effectively promulgating cancer cell destruction. Other important outstanding challenges include: (1) whether structurally modified flavonoids are equally effective in regulation of PD-L1 expression, (2) whether cancer cell types display similar or different sensitivity to modulation of PD-L1 by flavonoids, (3) nature of signaling pathways governing the control of PD-L1 by flavonoids, (4) the role of epigenetic control, specifically, histone acetylation/deacetylation, methylation/demethylation, in fine tuning the control of PD-L1 expression in response to flavonoids. We postulate that the combination of flavonoids may constitute a diet-based platform for modulating PD-L1 in the tumor microenvironment that has translational implications for enhancing patients’ response to PLB therapy. 

## 3. Induction of PD-L1 by ≥IC50 Supra-Pharmacological Grape Polyphenol Resveratrol and Piceatannol, Alone and Combined, in Different Cancer Cell Types 

In contrast to in vitro experiments on flavonoid polyphenols summarized in Table 1 showing that induced tumor PD-L1 is suppressed by pharmacologic-achievable concentration of flavonoids, we observed copious upregulation of PD-L1 in certain cancer cells by ≥IC50 supra-pharmacological dose of polyphenol resveratrol and piceatannol, alone and combined [19]. As mentioned above, resveratrol is a naturally occurring, non-flavonoid polyphenol present in various plant species and wine. In vitro and in vivo experiments have shown that resveratrol possesses a wide range of beneficial biological effects including anti-aging, anti-obesity, anti-inflammatory and anti-cancer activities. Chemoprevention by resveratrol was first reported using breast cancer models [14]; its clinical efficacy was subsequently validated in clinical trials by oral feeding of resveratrol to colorectal cancer patients [43,44]. 

Recently, in our published study, we first screened changes in PD-L1 expression by dietary polyphenols using human HCT116 colon cancer cells treated for 48 h with increasing concentration (5, 20 and 50 µM) of resveratrol (Res), piceatannol (Pic), pterostilbene (PTS), trimethylstilbene (TriMRes) and myricetin. Surface expression of PD-L1 in control and treated cells was assayed by flow cytometry. The most significant increase was observed in cells treated with 50 µM resveratrol or piceatannol, by 2.1- to 2.9-fold, respectively [19]. Since the chemopreventive activity of resveratrol was first demonstrated in skin and breast cancer models [14], we further examined the effects of resveratrol and piceatannol, alone and combined using Cal51 breast cancer cells. Cells were incubated for 48 h with 10, 50 and 250 µM resveratrol or piceatannol alone, or as combination (containing 100 µM each of resveratrol plus piceatannol). Resveratrol or piceatannol as single agents caused a non-linear increase in PD-L1 evident by doubling in PD-L1 expression at 250 µM. Significantly, combination of both polyphenols, each at 100 µM resulted in >6.2-fold synergistic increase of PD-L1 [19]. These results showing that treatment by resveratrol or piceatannol in some breast and colon cancer cell lines was accompanied by a dose-related increase in PD-L1 expression [19]. 

The specificity of PD-L1 regulation by resveratrol or piceatannol was further tested using a panel of prostate cancer (PC3, DU145, LNCaP and 22Rv1) and melanoma (RPMI7951, SKMEL28, SKMEL5 and HT144) cell lines (Table 2). A complex concentration-related increase of PD-L1 was observed with either polyphenol, alone or combined (Table 2). In LNCaP and RPMI7951 cancer cells, combined resveratrol and piceatannol showed synergistic effects, resulting in 2.8- and 2-fold increase in PD-L1 expression compared to cells exposed to either polyphenol (Table 2). Taken together, these results support that dietary polyphenols, resveratrol or piceatannol, are singularly effective in inducing PD-L1. Comparatively, the combination of resveratrol and piceatannol acts synergistically, leading to a more pronounced induction of PD-L1 expression in selected cancer cells derived from different anatomical sites including breast, colon and prostate cancer cells, and melanoma.

## 4. Transcriptional/Epigenetic Regulation of Tumor PD-L1 by Resveratrol/Piceatannol Occurs by HDAC3/p300-Mediated NF-κB Axis, Distinct from Classical, IFN-γ-Mediated Jak/STAT Canonical Mechanism 

**Figure 1:**
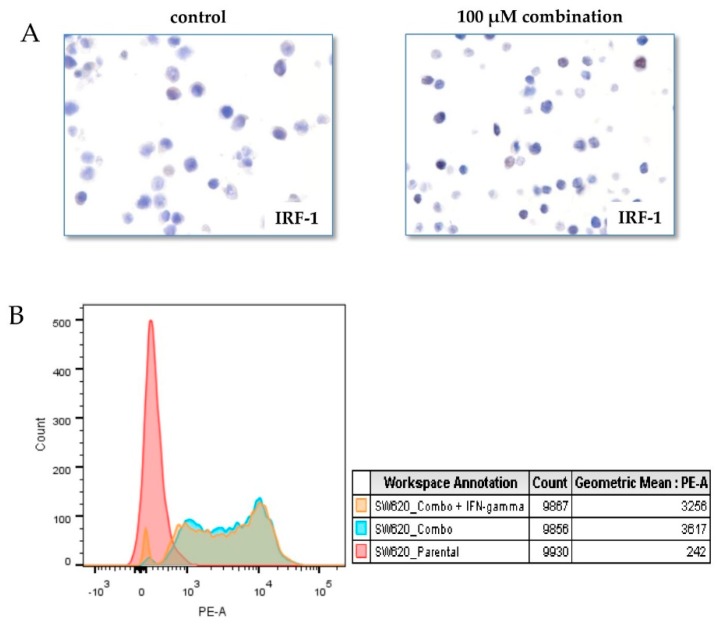
Effects of resveratrol and piceatannol on IRF (interferon regulatory transcription factor) and PD-L1 expression in SW620 colon cancer cells. Panel (**A**). Immunohistochemistry staining for IRF expression after treatment for 48 h with the 100 µM combination of resveratrol and piceatannol. No difference in IRF expression was observed in control (left) and treated cells (right). Images were captured at 20× and cropped to show cells representative of the effect of treatment. Panel (**B**). Colon cancer cell line was incubated for 48 h with 100 µM combination (combo) of resveratrol or piceatannol with or without IFN-γ. After treatment the cells were harvested and stained for PD-L1 expression before quantification by flow cytometry using the geometric mean fluorescent intensity of PE as the readout for expression of PD-L1. The values generated from flow cytometry were graphed to represent gains in PD-L1 expression.

Transcriptional control of PD-L1 by resveratrol or piceatannol was revealed in our recent published study using SW620 colon cancer cells [19]. The role NF-κB plays in resveratrol/piceatannol mediated PD-L1 induction was examined by NF‑κB reporter assays and immunohistochemistry analysis and data showing subcellular translocation and nuclear accumulation of the p65 subunit of NF‑κB. Further, since the increase in PD-L1 by resveratrol or piceatannol was attenuated by IKK inhibitor BMS-345541 and, in addition, histone post-translational modification inhibitors, respectively, resminostat, entinostat or anacardic acid, we suggest that regulation of PD-L1 is also under epigenetic control driven by HDAC3/p300-mediated NF-κB axis [19]. Classically, the induction of tumor PD-L1 expression occurs via IFN-γ-mediated Jak/STAT axis, under transcriptional activation of interferon regulatory transcription factor (IRF-1) [45,46]. Whether IRF-1 is involved in resveratrol/piceatannol mediated PD-L1 induction was tested in SW620 cells by immunohistochemical analysis. No change in IRF-1 occurred in cells after 48 h exposure to 100 μM combined treatment compared to control (Figure 1A). Thus, the upregulated expression of PD-L1 by combined resveratrol/piceatannol treatment in tumor cells tested is primarily governed by the NF-κB axis through an IFN-γ-independent mechanism. Since resveratrol induced IFN-γ expression in CD8+ T cells had been previously reported [17] and IFN-γ is a known inducer of PD-L1, we examined whether changes in surface expression of tumor PD-L1 by combined resveratrol/piceatannol might be enhanced by the addition of IFN-γ. No additional elevation of tumor PD-L1 occurred with the co-administration of IFN-γ and combined resveratrol and piceatannol, compared to cells exposed to 100 μM combined resveratrol and piceatannol (Figure 1B). 

## 5. Tumor PD-L1 Induction Is Concurrent with Emergence of Drug Resistance

Also of clinical relevance is whether evasion of host immune response by upregulation of PD-L1 may be in part linked to the emergence of drug resistance. To this end, we compared cell survival status using SW620 colon cancer cells treated for 48 h with combination of resveratrol and piceatannol, each at 100 μM (≥IC_50_), with untreated control SW620 cancer cells. We showed an increase in expression of γH2AX and cleaved caspase 3—both diagnosis of DNA fragmentation and damage as well as the induction of apoptosis. Further, we found down regulation of oncogenes p38-MAPK and c-Myc [19]. These results raise the possibility that ≥IC50 supra-pharmacological combined dose of polyphenols, by suppressing oncogenes required for tumor cell survival, as well as by causing DNA damage and induction of apoptosis, may trigger DNA instability and DNA damage response (DDR), impacting on the viability and vitality of tumor cells and tumorigenesis. As an alternative consideration, timely elimination of apoptotic cancer cells harboring damaged DNA, refractory to the genetic repair mechanisms, could give rise to accompanying genome-wide tolerance and lesion-prevalent cell survival, marked by the generation and subsequent expansion of residual, chemical-resistant cell populations. This scenario is plausibly aligned with our data showing that in certain cultured cancer cells, ≥IC50 polyphenols not only result in high upregulated PD-L1, but also activate NF-κB signal cascades which are actively required for mediating the induction of PD-L1 and for tumor cells to proliferate and thrive [19]. We propose that certain ≥ IC50 supra-pharmacological polyphenol-surviving cancer cells may be considered as a preclinical drug resistance model for dissecting the sequence of cellular and molecular events connecting the relationship between high PD-L1 and its added role in facilitating tumor cell survival, conferring resistance to chemotherapy, and host immunity.

## 6. Induction of PD-L1 by Resveratrol and/or Piceatannol May Serve as a “SEE (Search, Enhance, and Engage) Signal for Anti-PD-1/PD-L1 Immune Checkpoint Blockade, PLB

**Figure 2:**
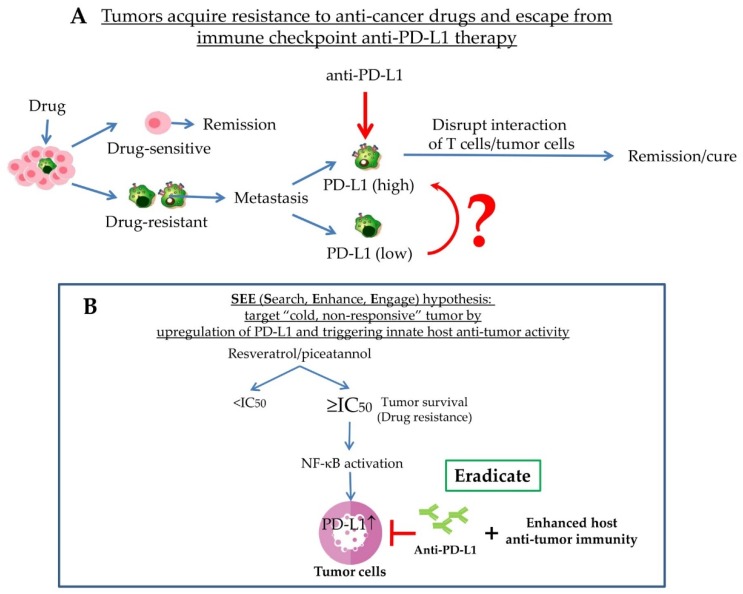
Schematic illustrating (**A**) mechanisms by which tumors acquire resistance to anti-cancer drugs and escape from immune surveillance and (**B**) “cold, non-responsive” tumor can display improved recognition and sensitization to anti-PD-L1 blockade monoclonal antibodies after treatment by ≥IC50 polyphenols that result in upregulation of tumor PD-L1. Specifically, this approach enhances the “visibility/recognition/identification” of low PD-L1-expressing tumors by combining PD-L1 inducers, for example, polyphenol resveratrol or piceatannol with PD-L1 immune checkpoint blockade antibodies. As hypothesized, induction of PD-L1 by resveratrol and/or piceatannol, occurring via NF-κB, serves as a sensitizing **S**earch, **E**nhance, and **E**ngage (“SEE”) signal for anti-PD-1/PD-L1 immune checkpoint blockade, PLB and is followed sequentially by the repertoire of innate host anti-tumor activity and response that effectively results in the eradication of tumor cells.

Our overarching goal in studies of dietary polyphenols are to test whether they might unravel novel biomarkers and discover complimentary therapeutic leads in the management of cancer patients treated by monoclonal antibodies that disrupt the interaction between PD-1/PD-L1, an approach known as PLB (immune checkpoint inhibitor therapy). PLB may be particularly applicable to individuals who have been declared in remission and now present metastatic, drug-resistant relapse. Because identifying erasable and therapy-resistant tumor clones can be difficult, and since predicating when cancer might recur in “cured” subjects equally lacks precision, upregulation of PD-L1 may be viewed as a reliable, assayable theranostic and mechanistic biomarker. Indeed, the FDA approves immunotherapy for patients who face recurrent cancer or fail prior lines of therapy and whose tumor expresses PD-L1. As such, the advent of PLB has made it possible to achieve tumor eradication, disease remission and cure, largely based on the premise that PLB mainly identifies immune escaped cancer cells showing positive membrane PD-L1 expression, making cancer less likely to return. As noted previously, the existence of tumor cells with no/low intrinsic surface PD-LI remains a problem, limiting the life-prolonging efficacy of PLB in patients to a relatively small percentage and equally important, a low clinical response rate [3,4,5,6,8,9,10,11,12,13]. We hypothesize that if tumor cells with no/low intrinsic surface PD-L1 can be selectively elevated, the efficacy or response to PLB may be significantly enhanced by better visibility. Studies testing this hypothesis led to the serendipitous discovery that surface PD-L1 in low PD-L1 expressing tumor cells can be markedly upregulated following treatment by supra-pharmacological dose of stilbenoids, viz., combined resveratrol and piceatannol. Our pursuit of effects of polyphenols on PD-L1 is further guided by the observation that in certain cancer cell types, high level of expression of PD-L1 in tumor cells is correlated with clinical efficacy of PLB [8,9,10,11,12,13]. Disease- and progression-free survival to PLB has been documented in melanoma, lung, renal and breast carcinomas [3,4,5,6], albeit in only a small percentage of patients, marked by prominent-PD-L1 and not negligible-PD-L1 tumors. In summary, while an increase in tumor PD-L1 will, in principle, promulgate evasion from immune surveillance by inactivating host T cells and hence is bad for tumor outcome [47,48,49] by favoring tumorigenesis, the potential negative impact of elevated PD-L1 is countered by the clinical efficacy PLB shows in patients expressing high PD-L1 in their tumors. In other words, the acquisition of drug-resistance and escape from host immune surveillance in tumors with high PD-L1 in such patients can be circumvented and therapeutically targeted by PLB. We therefore postulate that induction of tumor surface PD-L1 by stilbenoids is concomitant with the reorganization of the tumor microenvironment and may serve as a Search, Enhance, and Engage (“SEE”) signal to sensitize low PD-L1-expressing “cold, non-responsive” tumors, augmenting their detection by PLB and elimination by host anti-tumor immunity. It is our hypothesis that PD-L1 expressed by tumor, clinically used to select anti-PD-1/PD-L1 PLB-responders and functionally regarded as a “stop sign” to inactivate T cells, may have a “find me” role for anti-PD-1/PD-L1 PLB-non-responders, for ultimate elimination by the host anti-tumor immunity. Moreover, agents (dietary phytochemicals and/or chemotherapeutic agents, at supra-pharmacological doses) capable of upregulating PD-L1 is hypothesized be optimally used as a combination regimen, together with PLB, either concurrently, intermittently or sequentially and not as a standalone application which would be detrimental for tumor outcome. This is a paradigm shift and advances the field of immune-oncology. Feasibility of this hypothesis can be validated experimentally since tumors with low/undetectable PD-L1 can be treated with ≥IC50 supra-pharmacological polyphenols, resveratrol or piceatannol, concurrently or intermittently with, preceded by, or after the administration of PLB (Figure 2). 

## 7. Perspectives

Analyses of the cancer cell microenvironment in recent years using both murine models and in humans have identified membrane ligands and receptors in both tumor cells and infiltrating immune cells, as playing an integral role in the anti-tumorigenic immune response. Contributing to the immune response is the interplay between specific ligands and their cognate receptors acting to control signaling pathways linked to immune response. Among the most clinically relevant of those immune responsive ligand: receptor interactions is the ligand PD-L1 that targets immune surveillance by binding the receptor PD-1 on activated T cells. PD-1 and PD-L1 blocking antibodies have shown significant therapeutic impact in the treatment of melanoma, lung, renal and breast cancers [3,4,5,6]. Despite these advances, current diagnosis and treatment strategies of malignancies do not adequately reflect pathogenetic mechanisms and rational treatment targets. Accordingly, mechanistic details on the expression of immune-regulatory proteins such as PD-L1 are of clinical relevance and interest. Similarly, there is a lack of understanding and appreciation on whether and how the level of PD-L1 can be modulated by small molecules, particularly those that are ingested in the diet. These considerations provide the impetus for this mini-review. 

The recent interest on PD-L1 may be largely attributed to its apparent success in serving as a biomarker for cancer patient selection in immuno-oncology therapy, best exemplified by the use of PLB. Therefore, insights on PD-L1 regulation in clinical settings in general and specific cancer types is imperative, for enhancing efficacy and enlarging the therapeutic scope of existing PLB, for future development of PLB, and for rationally tackling many unresolved questions in PLB. Some aspects of regulation of PD-L1 and its biological functions in several cancer cell types and cancer cell microenvironment have been uncovered in recent years—from its genetic organization, epigenetic, transcription, post-transcription control and signaling [50,51,52,53,54,55]. Additional research gaps open to scientific and clinical investigations include: What genomic and non-genomic factors underlie or contribute to induction and hyper-expression of PD-L1 in tumor cells in the evolving stages of carcinogenesis? Are there different forms or subcellular pools of PD-L1 that can more reliably predict progression-free clinical response to PLB? Can PD-L1-unrelated molecular and biochemical profiles be developed to confidentially and differentially categorize “PLB-responders? What immunogenic characteristic profiles signify the “PLB-non-responders”? 

We summarize in this review that, using colon and breast cancer cells, ≥IC50 supra-pharmacological dose of grape polyphenols resveratrol and piceatannol, alone and combined, can upregulate PD-L1, activate NF-κB, and induce DDR. By contrast, pharmacological dose of flavonoids polyphenols, such as, curcumin, apigenin, EGCG and baicalein, are devoid of PD-L1 modulatory effects when used alone, but suppress the induction of PD-L1 in a variety of cancer cell types treated with either IFN-γ or EGF. This dichotomous observation is unexpected and may have complex mechanisms that relate to their dose-dependent and differential actionable mechanisms. We propose that upregulation of tumor PD-L1 induced by exposure to resveratrol/piceatannol may serve to flag or sensitize the tumor cells by enhancing “tumor cell visibility” for targeting by PLB, whereas, by contrast, suppression of tumor PD-L1 by flavonoid polyphenols may act to attenuate “tumor cell visibility” thereby reducing the therapeutic dose of PLB required and, in addition, counteract the side effects of PLB. Our hypothesis implies that beneficial response rate to PLB may be enhanced or attenuated when combined with dietary polyphenols. We further postulate that tumor cells with low/no PD-L1 can become active responders and that therapeutic efficacy to anti-PD-1/PD-L1 PLB can be improved via upregulation of PD-L1. In addition, we surmise that ≥IC50 supra-pharmacological dose of grape polyphenols resveratrol and piceatannol can induce DDR by targeting genotoxic NF-κB thereby impacting NF-κB mediated drug resistance as well as host-mediated anti-tumor immunity in the tumor microenvironment. Of note, an upregulation of PD-L1 has been observed post-anti-androgen therapy and X-ray irradiation [56,57], suggesting that patients subjected to such therapeutic intervention ought to benefit from PLB. For example, a 2018 article points to the enhanced antitumor effects combining radiation with PD-1/PD-L1 blockade therapy [58]. The authors indicated that the proposed synergy mechanisms undermining the use of radiation therapy and PD-1/PD-L1 inhibitors occur in concordance with the modulation of the immune parameters within the tumor microenvironment including the upregulation of PD-1/PD-L1 in immune and tumor cells. It is noteworthy that the majority of cancer patients are treated with genotoxic, DNA-damaging chemicals prior to PLB; thus, our hypothesis advancing the understanding of the PD-L1, may unravel other molecular/immunologic changes applicable to the treatment of PLB-non-responders using combination therapy. Whether chemotherapeutic chemicals induce changes in tumor PD-L1 thereby establishing a new threshold for contributory effective response to PLB, remains to be tested.

## Figures and Tables

**Table 1:** Modulation of PD-L1 by flavonoids and polyphenols. A PubMed search using the input of “resveratrol and PD-L1” or “flavonoids and PD-L1” resulted in 13 hits. The studies showing the most definitive results and conclusions are presented in Table 1.

Polyphenol & System Tested	Structure	Results Observed	Reference
Curcumin: human melanoma & dendritic cells	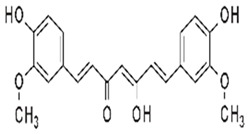	25 µM curcumin significantly inhibits IFN-γ (10 ng/mL) mediated induction of PD-L1 in 3 melanoma cells tested; same dose curcumin moderately inhibits IFN-γ (1000 U/mL) mediated induction of PD-L1 in PBMC-derived dendritic cells from healthy volunteers	[34]
Apigenin: human melanoma & dendritic cells	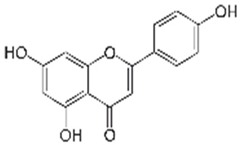	30 µM apigenin more effective than curcumin in inhibiting IFN-γ (10 ng/mL) mediated induction of PD-L1 in melanoma cells tested; apigenin highly effective in inhibiting IFN-γ (1000 U/mL) mediated induction of PD-L1 in PBMC-derived dendritic cells from healthy volunteers	[34]
EGCG: human NSCLC cells	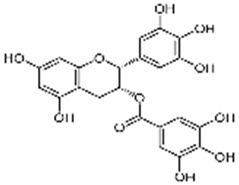	10 and 50 µM EGCG inhibits IFN-γ (10 ng/mL) mediated induction of PD-L1 by ~60–80% in A549 cells; 50 µM EGCG inhibits EGF (10 ng/mL) mediated induction of PD-L1 by ~50% in Lu99 cells	[35]
Baicalein: hepatocellular cells	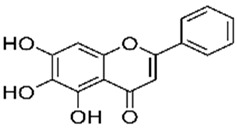	2.5, 5.0 and 10 µM baicalein dose-dependently inhibits IFN-γ (10 ng/mL) mediated induction of PD-L1 by ~60–70% in SMMC-7721 and HepG2 cells	[36]
Resveratrol ± piceatannol: breast and colorectal cells	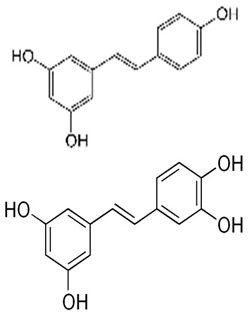	Dose-dependent upregulation of PD-L1 by resveratrol and piceatannol differs in the cell lines tested. The combination of resveratrol and piceatannol acts synergistically resulting in significant induction of PD‑L1 expression; specifically, ≥4.5-fold in Cal51 breast cancer and ≥3.5-fold in SW620 colon cancer cells, compared to 50 µM of either stilbenoid added alone	[19]

**Table 2:** Changes in level of expression of cell-surface PD-L1 by resveratrol/piceatannol alone or in combination (fold).

Cell Line Name	Indication	Resveratrol		Piceatannol		Combination	
		10 µM	50 µM	10 µM	50 µM	20 µM	100 µM
PC3	Prostate	0.8	1.3	0.6	1.1	1.7	1.4
DU145	Prostate	1.0	1.4	0.9	1.2	1.6	1.9
LNCaP	Prostate	1.2	1.3	1.8	1.4	1.7	2.8
22Rv1	Prostate	1.3	1.2	1.3	1.4	1.4	1.6
RPMI7951	Melanoma	0.8	1.4	1.0	1.1	1.6	2.0
SKMEL28	Melanoma	1.0	1.0	0.4	1.1	1.3	1.1
SKMEL5	Melanoma	1.0	1.1	0.5	1.2	1.3	1.1
HT144	Melanoma	1.3	1.4	1.1	1.6	1.4	1.5

The cells were cultured and assayed with the same protocols as described in Figure 1. The fold difference illustrates the treatment effects on PD-L1 against the untreated control. Induction defined as >1.3 fold increase.

## Abbreviations

CD4+ T cells	T helper cells
CD8+ T cells	cluster of differentiation 8, cytotoxic T cells
DDR	DNA damage response
HAT	histone acetyl transferase
HDAC3	histone deacetylase 3
γH2AX	gamma H2A histone family member X
IC_50_	the half maximal inhibitory concentration
IFN-γ	interferon gamma
IKK	inhibitor of nuclear factor kappa-B kinase
IRF-1	interferon regulatory transcription factor
Jak	Janus Kinase
p38-MAPK	p38 mitogen-activated protein kinase
c-Myc	a family of regulator genes and proto-oncogenes that code for transcription factors
MDSCs	myeloid-derived suppressor cells
NF-κB	nuclear factor kappa-light-chain-enhancer of activated B cells
p300	Histone acetyltransferase, also know p300 HAT
PD-1	Programmed cell death 1
PD-L1	Programmed cell death ligand 1
PE	Phytoerythrin
Pic	Piceatannol
PLB	PD-L1 immune checkpoint blockade
PTS	Pterostilbene
Res	Resveratrol
SEE	Search, Enhance, and Engage
STAT	signal transducer and activator of transcription protein
TriMRes	Trimethylstilbene
X-ray	X-radiation
